# Lattice dynamics of Ga_1−*x*_Mn_*x*_N and Ga_1−*x*_Mn_*x*_As by first-principle calculations

**DOI:** 10.1186/1556-276X-7-573

**Published:** 2012-10-17

**Authors:** Horacio W Leite Alves, Luísa MR Scolfaro, Eronides F da Silva

**Affiliations:** 1 Departamento de Ciências Naturais, Universidade Federal de São João del Rei, Praça Dom Helvécio, 74, São João del Rei, 36301-160, MG, Brazil; 2 Department of Physics, Texas State University, San Marcos, TX, 78666, USA; 3 Departamento de Física, Universidade Federal de Pernambuco, Cidade Universitária, Recife, PE, 50670-901, Brazil

**Keywords:** Dilute nitride semiconductor, Phonons, VCA model, DFT calculations, Nanostructures

## Abstract

In this work, we present theoretical results, using first-principle methods associated to the virtual crystal approximation model, for the vibrational mode frequencies of both the Ga_1−*x*_Mn_*x*_N (in both cubic and hexagonal structures) and the Ga_1−*x*_Mn_*x*_As alloys, with the Mn contents in the range of 0% to 20%. The dependence of the calculated phonon frequencies with the Mn content was analyzed, and the results indicate that the phonon frequencies decrease with the increasing of Mn composition, leading to the false impression that they obey the Vegard rule in some cases. Moreover, the hexagonal Ga_1−*x*_Mn_*x*_N alloys are elastically unstable for Mn concentrations at the order of 20%, which explains in part the experimentally observed deterioration of these alloys. These findings can be used in future technologies as a guide for the synthesis of spintronic nanostructured devices, such as nanowires, based on these materials.

## Background

Over the last decade, extensive studies of dilute magnetic semiconductors (DMSs) and oxides have confronted research with experimental and theoretical challenges that make this field as one of the most exciting and controversial in the condensed matter theory. In particular, semiconductors, such as GaN and GaAs, doped with magnetic impurities are highly promising candidates for DMSs to realize ferromagnetism above room temperature [1,2]. Also, due to the miniaturization of electronic devices to the nanoscale, nanostructures such as quantum wells, quantum dots and nanowires (NWs) that are controlled grown, are a very important class of new semiconductor-based spintronic nanodevices [3].

However, most of the grown DMS GaN-based NWs have diameters which vary from 10 to 100 nm [3]. In this range, recent theoretical results show that their calculated electronic properties tend to the values of their respective bulk counterpart [4,5]. Moreover, measured GaN NW phonon frequencies show the same features [6], and NW surface effects play an interesting role in the electronic bandgap formation and the appearance of some phonon modes [4-6].

Despite the fact that Raman and infrared techniques were widely used in the last decade to characterize DMS materials as Ga_1−*x*_Mn_*x*_N and Ga_1−*x*_Mn_*x*_As [7-10], theoretical data on their phonon modes available in the literature are very scarce. However, there are at least two approaches to describe the vibrational modes of an alloy: the virtual crystal approximation (VCA) and the percolation model [11]. In the VCA model, each atom *A* is surrounded by four virtual *B*_1−*x*_*C*_*x*_ atoms, i.e., the crystal is viewed at the macroscopic scale as a continuum or, in a well-ordered alloy, the physical properties are accordingly averaged. Thus, VCA provides, at first sight, a good start in the description of many vibrational properties in semiconducting alloys.

From the experimental point of view, it is interesting to remark that, with the increase of Mn content, both the Ga_1−*x*_Mn_*x*_As longitudinal optical (LO) and transverse optical (TO) phonon frequencies decrease. Besides, in the molecular beam epitaxy grown samples, a local Mn mode vibration was not observed, as observed in the ion-implanted samples [9,10]. For the Ga_1−*x*_Mn_*x*_N alloys, it was verified that the *A*_1_(LO) mode is shifted to higher frequencies, while the *E*_1_(LO) mode decreases with the increasing Mn content. Also, at concentrations around 12% of Mn, rapid deterioration in the lattice ordering occurs due to Mn precipitation [8,12]. For that, any theoretical study on Ga_1−*x*_Mn_*x*_N and Ga_1−*x*_Mn_*x*_As materials should address these points.

Therefore, in this work, we calculate using first-principle methods together with the VCA model the frequencies of the vibrational modes for both the Ga_1−*x*_Mn_*x*_N and Ga_1*−x*_Mn_*x*_As alloys, with Mn contents varying up to 20%. From the obtained results, the dependence of the calculated phonon frequencies with the Mn content is analyzed.

## Methods

### Theoretical model

Calculations were carried out using DFT within both the local density approximation (LDA) and the generalized gradient approximation (GGA) for the exchange-correlation term, plane-wave description of the wave functions and the pseudopotential method (ABINIT code [13]). We have used the Troullier-Martins pseudopotentials, and the phonons were obtained by means of the density-functional perturbation theory. The results were converged with a cutoff energy of 120 Ry of the plane wave expansion of the wave functions, and we have used a (6, 6, 6) Monkhorst-Pack mesh to sample the Brillouin zone. In order to describe the alloys, we have used the average crystal approximation (within the VCA model), as defined by Ghosez et al. [14]. For each Mn composition *x* of the alloy within the VCA model, the total energy of the respective unit cell was minimized as a function of both the lattice parameters and the atomic positions by means of conjugated gradient techniques. We would like to remark that both the structural parameters and vibrational modes of the isolated MnAs, MnN, GaMnAs, and GaN compounds were calculated and described in our previous works [15-17].

Concerning the calculated structural properties for the cubic Ga_1−*x*_Mn_*x*_As alloys at the diluted regime, we have shown that the unit cell expands with the increase of the Mn content *x*[16]. For lower Mn concentrations (*x* < 5%), this expansion is linear, which is in agreement with the experimental findings [18], and it reaches a maximum at *x* ≈ 10% (15%) in the LDA (GGA) results. Also, there is a lattice contraction with the increase of the Mn content, and this is observed in both LDA and GGA calculations. For both the cubic and hexagonal Ga_1−*x*_Mn_*x*_As alloys (not shown here), the same trends were observed: in both structures, the lattice expansion reaches its maximum at *x* ≈ 15%, which is independent of the exchange-correlation term used in the calculations. Moreover, it is interesting to remark that the *c*/*a* ratios of the hexagonal structures remain constant with the Mn content variation.

## Results and discussion

**Figure 1:**
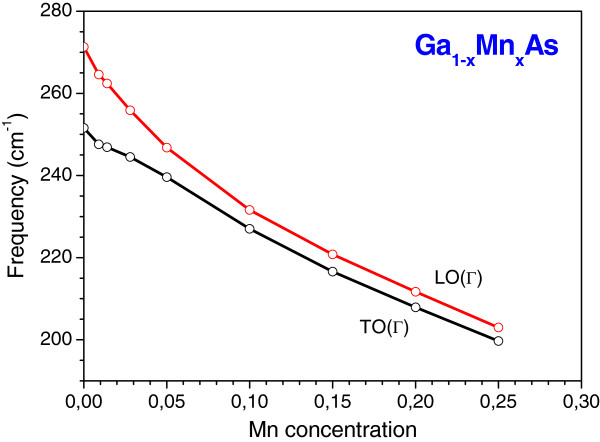
**Calculated LO(Γ) and TO(Γ) phonon frequencies for cubic Ga**_**1−*x***_**Mn**_***x***_**As alloy as function of Mn concentration.**

**Figure 2:**
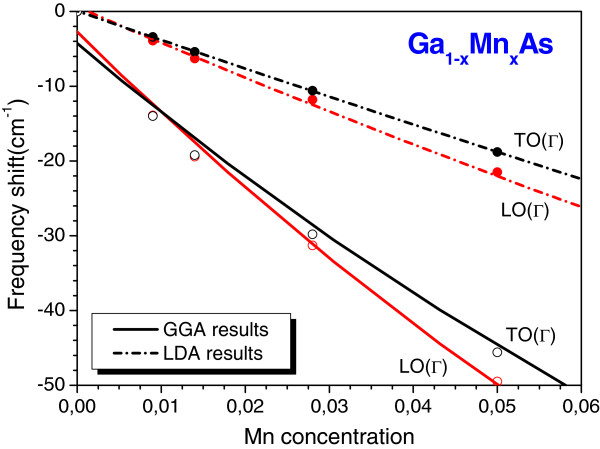
**Calculated GGA and LDA LO(Γ) and TO(Γ) frequency shifts for the cubic Ga**_**1−*x***_**Mn**_***x***_**As alloy, as function of Mn concentration (diluted regime).** Calculated GGA (open circles and full lines); and LDA (full circles and dash-dot lines). The full lines (obtained by a second degree polynomial) are guides for the eyes.

Figure 1 shows the calculated GGA LO(Γ) and TO(Γ) phonon frequencies and its dependence on the Mn concentration *x* for the cubic Ga_1−*x*_Mn_*x*_As alloys. As observed in our previous LDA results [17], the frequencies of these two modes diminish with the increasing Mn composition. We have also noted that for *x* < 5%, the LO(Γ) mode is more sensitive to the changes in the Mn concentration than the TO(Γ) mode. This may be more clearly seen in Figure 2, wherein we display both our calculated GGA and LDA (taken from the study of HWLA [16]) frequency shifts (Δ*ω*) as a function of the Mn composition for these two modes. Despite the fact that our results show that the frequency shifts are quite sensitive to the exchange-correlation term used to evaluate the phonons for this alloy, these are consistent with the experimental findings [10].

In Figure 2, the obtained GGA frequency shifts of both LO(Γ) and TO(Γ) modes were fitted by second-degree polynomials, with a strong positive curvature. From the fitting, we obtained the following:

$$\Delta \omega_{\text{TO}}=1,509x^{2}-301x-0.7\text{,}$$

for the TO mode, and

$$\Delta \omega_{\text{LO}}=3,344x^{2}-646x-0.4\text{,}$$

for the LO one. As a consequence, our results indicate that for the phonon frequencies, this material does not obey the Vegard rule. We would like to point out that our evaluated LO(Γ) and TO(Γ) phonon frequencies for MnAs (*x* = 100%) in the zincblende structure are 328.8 and 272.1 cm^−1^, respectively [17]. In order to obey the Vegard rule, these mode frequencies should have a positive linear dependence on the Mn content, which is not observed in our results.

However, when we compare our results for the LO(Γ) and TO(Γ) frequency shifts with the experimental data [10], the agreement is only qualitative once our GGA results are 40% lower than the experimental data. We can attribute this discrepancy to the fact that Mn induces local distortions in the GaAs lattice [19], giving rise to local modes, which were detected only in ion-implanted sample experiments [9], and it is not predicted by the VCA model. We would like to point out that in the VCA model, no singularity is expected on the TO modes, and then, the TO frequencies shift regularly between the natural frequency in the pure crystal and the impurity frequency [11]. Moreover, the most stable structure of MnAs is the hexagonal NiAs modification [17], for which the local environment of the Mn atoms is quite different from that found in the zincblende structure. This means that the Ga_1−*x*_Mn_*x*_As-diluted alloy is not well ordered as required in the VCA model.

**Figure 3:**
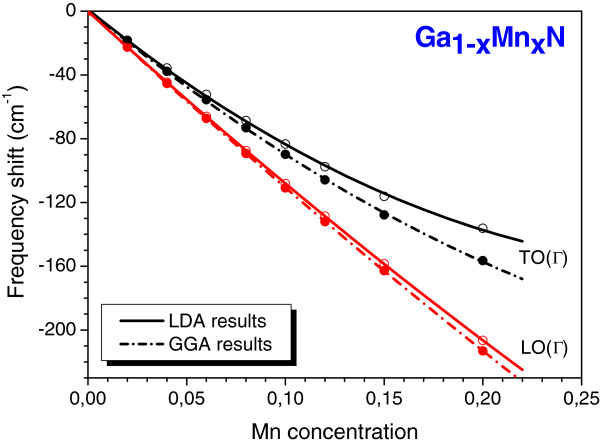
**Calculated GGA and LDA LO(Γ) and TO(Γ) frequency shifts for the cubic Ga**_**1−*x***_**Mn**_***x***_**N alloy, as function of Mn concentration (diluted regime).** Calculated GGA (full circles and dash-dot lines) and LDA (open circles and full lines). The full lines (obtained by a second-degree polynomial) are guides for the eyes.

However, for the cubic Ga_1−*x*_Mn_*x*_N alloys, the nature of the local environment of the Mn atoms in this structure is quite different. We show in Figure 3 our calculated GGA and LDA frequency shift (Δ*ω*) dependence on the Mn composition for these two modes, LO(Γ) and TO(Γ). It is clear from Figure 3 that the same features observed in the GaMnAs case are also verified for this case, but in this case, the frequency shifts are not too sensitive to the exchange-correlation term used to evaluate the phonons. Here, the polynomial fitting gives the following:

$$\Delta \omega_{\text{TO}}=1,549x^{2}-1,002x+1.1\text{,}$$

for the LDA TO mode, and

$$\Delta \omega_{\text{LO}}=492x^{2}-1,130x-0.1\text{,}$$

for the LDA LO one. In this case, the decreasing LO mode frequency is stronger than that observed for the Ga_1−*x*_Mn_*x*_As alloys. Considering that the calculated LO(Γ) and TO(Γ) modes are at 710 and 534 cm^−1^, respectively, for GaN [15] and at 666.5 and 506.6 cm^−1^, respectively, for the zincblende MnN [17], one should expect that this alloy obeys the Vegard rule. However, the large curvature obtained rules out this fact.

Our previous calculations for MnN show that this compound in the zincblende structure is not stable [17]. In fact, MnN is more stable in the face-centered tetragonal structure, in which, as seen for the Ga_1−*x*_Mn_*x*_As case, one should also expect that Mn induces local distortions in the GaN lattice, similar to those verified for the Mn impurity in GaN [19], giving rise to local vibration modes. However, in the most stable structure, our previous results show that MnN has two modes: an *E*_u_ at 251.6 cm^−1^ and an *A*_2u_ at 330.4 cm^−1^ (both infrared active only). As we did not find any trace of these modes in our results for the cubic Ga_1−*x*_Mn_*x*_N alloys, we can conclude that based on our findings, the structural properties of these diluted alloys prefer to remain in the zincblende structure.

It is interesting to remark that our phonon frequency results for both the cubic Ga_1−*x*_Mn_*x*_As and Ga_1−*x*_Mn_*x*_N alloys show that we have a two-bond to one-mode character (a mixed mode) in these compounds, i.e., a ‘one-mode’ behavior. Thinking about the mass differences between Ga and Mn atoms, the obtained results show that it would be difficult to obtain a ‘two-mode’ behavior as that verified in our previous calculations for the Al_*x*_Ga_1−*x*_N alloys [20]. However, considering the position of the Mn impurity mode in the percolation model developed by Pagès and co-workers [11],

(1) $${\left(\omega_{\mathrm{TO}}-\omega_{\mathrm{Mn}}\right)}^{2}=\left[\frac{-6\gamma_{\mathrm{TO}}\Delta \ell }{\ell }\right]\omega_{\mathrm{TO}}^{2}\text{,}$$

where γ_TO_ is the Grüneisen parameter of the bulk TO mode, Δℓ is the alloy cation-anion bond-length difference related to the bulk one ℓ, and *ω*_TO_ is the bulk TO phonon frequency, as our calculated structural results show that all the considering lattices expand in the diluted regime; an imaginary value for the phonon frequency of the Mn impurity mode is found in all considered cases. This supports the observed one-mode character in our results.

**Figure 4:**
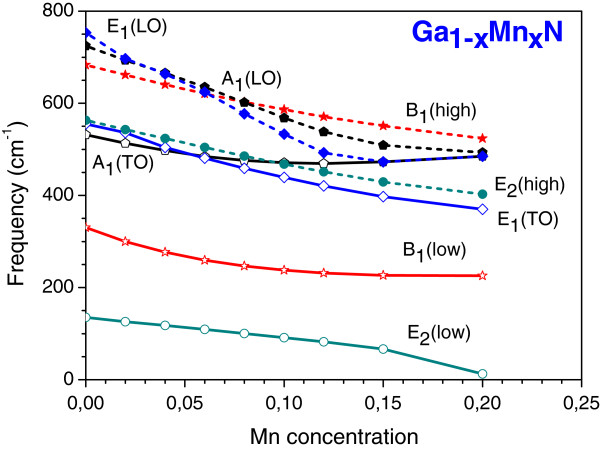
**Calculated phonon frequencies for the hexagonal Ga**_**1−*x***_**Mn**_***x***_**N alloy as function of the Mn concentration.**

In Figure 4, we show the calculated phonon frequency dependence on the Mn concentration for the hexagonal Ga_1−*x*_Mn_*x*_N alloy. From this figure, we note that, except for the *A*_1_(TO) phonon, all the other modes have their frequencies decreasing with the increase of Mn content. These findings are, qualitatively, in good agreement with the experimental data [8,12] and also follow the same features obtained for cubic alloys. Additionally, from the results depicted in Figure 4, we observe that the *E*_2_(low) mode became softer with the increase of Mn concentration, and when this content is greater than 20%, this mode has a negative frequency. From the lattice dynamics point of view, this is a clear indication that there is an elastic instability of the hexagonal Ga_1−*x*_Mn_*x*_N alloy, leading to some structural phase transition, as observed for the cubic HfO_2_ and ZrO_2_ compounds [21]. This is an indication to explain the deterioration in the Ga_1−*x*_Mn_*x*_N lattice [7]. Moreover, as the obtained *E*_2_(low) mode frequency becomes 135.1 cm^−1^, a value close to *E*_u_ which is one of the face-centered tetragonal MnN mentioned above, we can infer that this deterioration can be assigned to the rearrangement of the N atoms bonded to Mn that change the local symmetry around the metal, from the hexagonal to the tetragonal (or cubic) structure. These N atom rearrangements are increased as the Mn content increases, leading to the observed deterioration of the hexagonal lattice. However, as the crystal is viewed in a well-ordered alloy in the VCA model, our current results cannot describe, accurately, this observed deterioration. Thus, a good physical picture of this effect can only be described using total energy and phonon mode calculations where the local environment of the Mn atoms is taken into account. Such calculations are currently under way.

## Conclusions

In summary, we have calculated, using first-principle methods together with the VCA model, the vibrational modes of both the Ga_1−*x*_Mn_*x*_N (in both cubic and hexagonal structures) and the Ga_1−*x*_Mn_*x*_As alloys, with the Mn contents varying in the range of 0% to 20%. Our results agree qualitatively well with the available experimental data, whenever these comparisons were possible. Based on these results, we have shown interesting aspects concerning the vibrational modes of these alloys: in the range of the available experimental data, the phonon frequencies decrease with the increase of Mn composition, leading to a false impression that they obey the Vegard rule. Due to the characteristics of the VCA model, their optical modes show a one-mode behavior. Additionally, our results indicate that the hexagonal Ga_1−*x*_Mn_*x*_N alloys are elastic, which is unstable for the Mn concentrations at the order of 20%, and that explains, in part, the experimentally observed deterioration of these alloys. We reason that these findings can give guidelines for future nanostructured material systems, leading to further experiments and calculations on this subject. This will be important in the development of new spintronic nanodevices based on these DMS materials.

## Competing interests

The authors declare that they have no competing interests.

## Authors’ contributions

HWLA conceived the study and carried out the calculations. HWLA, LMRS, and EFSJ discussed the results and proposed new calculations and improvements. All authors read and approved the final manuscript.
